# A new species of *Chromis* damselfish from the tropical western Atlantic (Teleostei, Pomacentridae)

**DOI:** 10.3897/zookeys.1008.58805

**Published:** 2020-12-31

**Authors:** Emily P. McFarland, Carole C. Baldwin, David Ross Robertson, Luiz A. Rocha, Luke Tornabene

**Affiliations:** 1 School of Aquatic and Fishery Sciences, University of Washington, Seattle, WA 98195-5020, USA University of Washington Seattle United States of America; 2 Burke Museum of Natural History and Culture, Seattle, WA 98105, USA Burke Museum of Natural History and Culture Seattle United States of America; 3 Department of Vertebrate Zoology, National Museum of Natural History, Smithsonian Institution, Washington, DC, 20560, USA Smithsonian Institution Washington United States of America; 4 Smithsonian Tropical Research Institute, Balboa, Republic of Panama Smithsonian Tropical Research Institute Balboa Panama; 5 Department of Ichthyology, California Academy of Sciences, San Francisco, California 94118, USA Department of Ichthyology, California Academy of Sciences San Francisco United States of America

**Keywords:** Caribbean, coral reef, mesophotic, phylogenetics, rariphotic, systematics

## Abstract

Initially described in 1882, *Chromisenchrysurus*, the Yellowtail Reeffish, was redescribed in 1982 to account for an observed color morph that possesses a white tail instead of a yellow one, but morphological and geographic boundaries between the two color morphs were not well understood. Taking advantage of newly collected material from submersible studies of deep reefs and photographs from rebreather dives, this study sought to determine whether the white-tailed *Chromis* is actually a color morph of *Chromisenchrysurus* or a distinct species. Phylogenetic analyses of mitochondrial genes cytochrome b and cytochrome c oxidase subunit I separated *Chromisenchrysurus* and the white-tailed *Chromis* into two reciprocally monophyletic clades. A principal component analysis based on 27 morphological characters separated the two groups into clusters that correspond with caudal-fin coloration, which was either known or presumed based on the specimen’s collection site according to biogeographic data on species boundaries in the Greater Caribbean. Genetic, morphological, and biogeographic data all indicate that the white-tailed *Chromis* is a distinct species, herein described as *Chromisvanbebberae***sp. nov.** The discovery of a new species within a conspicuous group such as damselfishes in a well-studied region of the world highlights the importance of deep-reef exploration in documenting undiscovered biodiversity.

## Introduction

*Chromisenchrysurus* Jordan & Gilbert, 1882 is a species of Pomacentridae found on reefs in the tropical and subtropical western Atlantic Ocean from 5–146 m depth (Emery and Smith-Vaniz 1982). The species was first described by Gilbert and Jordan (1882) based on three specimens from Pensacola on the northeast Gulf of Mexico coast of Florida, USA. Jordan later provided the etymology in ‘The Fishes of North and Middle America’ (Jordan and Evermann 1898) as *ἔνχρυσος* (enchrysos), meaning deep golden, and *ορὺά* (oura), meaning tail, indicating that the species was named for its bright yellow caudal fin. Studies dating back to at least Smith-Vaniz and Emery (1980) refer to this species as *Chromisenchrysura*, based on the fact that the genus *Chromis* is feminine (Emery 1975). However, Jordan and Gilbert (1882) did not specify whether *enchrysurus* was intended as an adjective or noun in apposition. Following article 31.2 of the International Code of Zoological Nomenclature, if it is unclear how the name was intended, the name should then be treated as a noun in apposition with the original spelling unchanged, and gender need not match that of the genus. Thus, the original name *enchrysurus* is retained.

Emery and Smith-Vaniz (1982) redescribed *C.enchrysurus* and analyzed the morphological variation between populations of the species across its range. They noted that *C.enchrysurus* occasionally possesses white instead of yellow on the caudal, pelvic, anal, and posterior portion of dorsal fins, and that the species comprises either two or three populations (Bermuda, Brazil and Caribbean plus USA) that are morphologically distinct. However, most specimens used in the study were not observed alive, so any correlation between caudal-fin color and morphology or location could not be determined. Furthermore, since no genetic data were available at that time, the white-tailed *Chromis* was assumed to be a color morph of *C.enchrysurus* that shared a geographic range and lacked significant differences in morphology (Emery and Smith-Vaniz 1982).

Some of the confusion around the distribution and general biology of the species stems from the white-tailed form being restricted to deep reefs at or below the lower boundary of conventional SCUBA diving (~ 40 m). However, research on deep-reef fishes has significantly expanded in the last decade due to advances in technical diving and the use of manned submersibles and remote operated underwater vehicles (ROVs) (Gilmore 2016; Baldwin et al. 2018a; Rocha et al. 2018). In the Caribbean, this has been driven largely by the Smithsonian’s Deep Reef Observation Project (DROP). DROP uses manned submersibles to document in-life coloration, collect fresh specimens, and observe live specimens in their natural habitat. Such initiatives have led to the discovery that Caribbean mesophotic (~ 40–130 m) and rariphotic (~ 130–300 m) fish communities are taxonomically distinct from their shallow reef counterparts (Baldwin et al. 2018a; Rocha et al. 2018) and contain a wealth of undescribed biodiversity (Baldwin and Robertson 2013, 2014, 2015; Baldwin and Johnson 2014; Baldwin et al. 2016a, b, 2018b; Tornabene et al. 2016a, b, c; Tornabene and Baldwin 2017, 2019). Similar efforts at sites across the central and western Pacific have resulted in the discovery of new deep-reef fishes in a variety of taxonomic groups, including the genus *Chromis* (e.g., Arango et al. 2019; Pinheiro et al. 2019; Tea et al. 2019). Many recently described deep-reef species had never been observed before; however, in some cases, individuals formally recognized as juveniles or color morphs of known species have been identified as new species through observations of fresh and live coloration of both juveniles and adults coupled with DNA analysis (e.g., Baldwin et al. 2016a). At the outset of this study, we considered that this may be the case for the two putative color morphs of *C.enchrysurus*.

To date, DROP researchers have made collections at five deep-reef sites spanning the eastern and western Caribbean and have documented various *Chromis* species at each site, including the white-tailed morph of *C.enchrysurus*. In addition, one of us (LAR) has recorded *Chromis* spp. from closed-circuit rebreather dives off oceanic islands and the coast of Brazil, south to São Paulo State. Through these observations and collections together with photographic records accumulated by Robertson and Van Tassell (2019), comparisons between the two color morphs of *C.enchrysurus* made it possible to evaluate whether they represent distinct species. We combine molecular data from the mitochondrial genes cytochrome b and cytochrome c oxidase I, color photographs, distribution data, and morphological data from specimens collected across the entire range of the species complex to demonstrate that the white-tailed color morph represents a distinct species of *Chromis*, which we describe here, that is largely allopatric with *C.enchrysurus*. The discovery of this species contributes to our growing understanding of underexplored deep-reef ecosystems.

## Materials and methods

### Geographic range estimation

To determine the geographic range for both color morphs we used data from Robertson and Van Tassell (2019), which includes georeferenced records based on a number of public data aggregators (i.e., OBIS [www.obis.org], GBIF [www.gbif.org], FishNet2 [www.fishnet2.net], iDigBio [www.idigbio.org]), museum specimen databases, and independent collection efforts from the authors and dozens of contributing photographers. Initial estimates of the location of each color morph were made based on the photo-verified records and eyewitness reports from contributors to Robertson and Van Tassell (2019). This was supplemented with data from literature surveys of ROV studies (Colin 1974, 1976; Luiz et al. 2008; Pinheiro et al. 2016; Rosa et al. 2016; Simon et al. 2016; Stefanoudis et al. 2019), and our own specimens collected/observed from DROP surveys and rebreather dives, creating more comprehensive range estimates. Gaps in data were inferred based on estimates derived from typical biogeographic breaks and provinces in the Greater Caribbean region as described by Robertson and Cramer (2014).

### Specimens

Four fresh yellow-tailed specimens of *Chromisenchrysurus* were collected from Marathon Key, Florida by Frank Young (Dynasty Marine, Inc; https://dynastymarine.net). Eleven fresh white-tailed specimens were collected from Curaçao and Sint Eustatius during submersible expeditions carried out by DROP. An additional eleven samples collected by DROP from Curaçao were represented only from tissue samples (vouchers were not retained), but white fins were noted from these specimens at the time of collection. DROP specimens were collected by the ‘Curasub’ crewed submersible, which was equipped with a quinaldine ejection system that was used to anesthetize the fish. A suction tube terminating in a holding tank was used to collect and retain the fish once sedated. Collections took place periodically from 2010 to 2019.

For parts of the species range where no fresh specimens were available, specimens were examined from the University of Kansas (**KU**), the Florida Fish and Wildlife Conservation Commission’s Fish and Wildlife Research Institute (**FSBC**), the University of Florida (**UF**), Louisiana State University Museum of Zoology (**LZUMZ**), and the Field Museum of Natural History (**FMNH**); collection acronyms follow Sabaj (2020). Livecoloration of preserved specimens was presumed based on estimated ranges of color morphs observed in georeferenced photographs. Eleven of the preserved specimens had associated tissue samples, allowing retroactive confirmation of color morph through genetic comparison with confirmed vouchers. Detailed information on specimens examined in this study is provided in Suppl. material 1: Table S1.

### Morphology

Morphological data were collected from 15 specimens of white-tailed morphs and 32 specimens of yellow-tailed morphs following methods of Pyle et al. (2008). We did not measure caudal fin concavity due to the condition of specimens. Nearly all characters used here were also analyzed by Emery and Smith-Vaniz (1982). A total of 28 characters were measured (plus standard length), and seven characters were counted. Measurements were taken using digital calipers to the nearest 0.1 mm, and counts were made with the aid of a Zeiss Discovery v20 SteREO microscope and cyanine blue dye (Saruwatari et al. 1997) when necessary. Vertebral counts are total vertebral elements (precaudal + caudal vertebrae) not including the urostyle, and were taken from six specimens, three of each color morph, using micro computed tomography scans taken on a Bruker Skyscan 1173 micro-CT scanner at the Karel F. Liem imaging facility at Friday Harbor Laboratories, University of Washington. Due to the condition of some specimens, certain measurements and counts could not be obtained.

Morphological data were analyzed using a Principal Component Analysis (PCA) conducted in RStudio (RStudio Team 2015) after converting values to residuals via linear regression to correct for variation attributable to specimen size. All 28 morphometric variables except standard length were included in the PCA. Average values of a measurement for color morphs were used for specimens that were missing a specific measurement due to condition (12 specimens were missing at least one measurement for a total of 20 data points).

### Molecular methods

DNA was extracted from tissue preserved in 95% ethanol using the Qiagen DNAeasy Blood and Tissue Kit (Qiagen, Valencia, California). For USNM specimens, DNA was extracted using an automated phenol:chloroform protocol on the Autogenprep965 (Autogen, Holliston, MA) using the mouse tail tissue protocol (Baldwin et al. 2009).

The mitochondrial gene cytochrome b (cytb) was targeted using primers Fishcytb-F and Trucytb-R (Sevilla et al. 2007). The mitochondrial gene cytochrome c oxidase I (COI) was targeted using FISHCO1LBC and FISHCO1HBC (Baldwin et al. 2009) or FishF-1 and FishR-1 (Ward et al. 2004). Both genes were amplified via PCR using GoTaq Hotstart Master Mix (Promega, Madison, Wisconsin) using thermal profile as described in Sevilla et al. (2007) and Weigt et al. (2012). Cytb amplification was successful for 24 specimens and COI amplification was successful for 23 specimens. Sanger sequencing was performed at MCLAB and Texas A&M University – Corpus Christi Genomics Core Facility.

Sequences were trimmed, aligned, and concatenated in Geneious version 10.2.6 (Kearse et al. 2012). Sequences for other members of the genus *Chromis* and outgroups were gathered from GenBank or sequenced from USNM samples (see Suppl. material 2: Table S2). The concatenated alignment consisted of 87 sequences representing 53 pomacentrid species and four genera. The cytb alignment consisted of 71 sequences representing 49 pomacentrid species and four genera. The COI alignment consisted of 41 sequences representing 19 species and four genera. All three alignments contain representatives of *Chromis* from the Atlantic, Pacific, and Indian oceans, in addition to eight species from three other genera as outgroups (*Chrysiptera*, *Dascyllus*, *Pomacentrus*).

Substitution models and codon-partitioning schemes for each gene were selected using PartitionFinder2 (Lanfear et al. 2016) on XSEDE (Towns et al. 2014) through CIPRES (Miller et al. 2010). Phylogeny was estimated using MrBayes version 3.2 (Ronquist et al. 2012) on XSEDE (Towns et al. 2014) through CIPRES (Miller et al. 2010). Bayesian phylogenetic analyses were run for individual gene alignments and concatenated alignment for burn-in periods of 10%. Resulting consensus trees with posterior probability were visualized using FigTree v1.4.4 (accessible at http://tree.bio.ed.ac.uk/software/figtree/). Genetic distance matrices for both within- and between-group distances for both gene alignments were calculated in MEGA-X (Kumar et al. 2018). Distance values were calculated as the average number of base differences per site over all sequence pairs between groups (uncorrected p-distance). Positions with less than 95% site coverage were eliminated from the analysis. The cytb analysis consisted of 63 nucleotide sequences representing 41 species, and 324 positions were used for the final calculations. The COI analysis consisted of 31 nucleotide sequences representing ten species, and 603 positions were used for the final calculations. The alignments are available on Dryad (https://doi.org/10.5061/dryad.h9w0vt4gr).

## Results

### Geographic range

**Figure 1.**
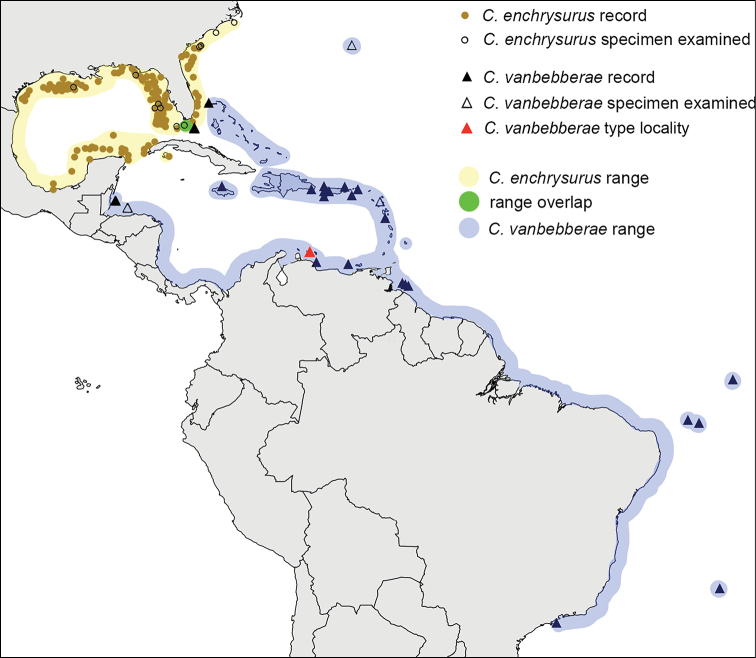
Observations and hypothesized ranges of *Chromisenchrysurus* and *Chromisvanbebberae*. Open circles and triangles represent locations of specimens examined in this study. Solid circles or triangles represent records from visual observations, database searches, or the literature. Red triangle is Curaçao, the type locality of *C.vanbebberae*.

Analysis of the geographic ranges of color morphs indicate little overlap between yellow- and white-tailed morphs (Fig. 1). The yellow-tailed individuals occupy the Gulf of Mexico to the eastern tip of the Yucatan Peninsula, western Cuba, Florida, and the U.S. southern Atlantic coast. This includes the species’ type locality, Pensacola, Florida (Jordan and Gilbert 1882). The white-tailed form occurs from the Bahamas, Bermuda and the Caribbean, south along the coast of South America to São Paulo, Brazil, and the Brazilian oceanic islands (Atol das Rocas, Fernando de Noronha, St. Paul’s Rocks, and Trindade), and was previously recorded in most of these locations as *C.enchrysurus* (Pinheiro et al. 2018). The exact boundary off Cuba between the two color morphs is uncertain due to limited data. There is an area of overlap in the Florida Keys near 24.785167, -80.6595 in which both color morphs occur but are segregated by depth: the yellow-tailed morph occurring in shallower water (~ 25–40 m), and the white-tailed morph occurring in deeper water (~ 60–90 m; Frank Young, Dynasty Marine, pers. comm). The extent to which this overlap extends up the US coast is unknown; to date, white-tailed individuals have only been observed in the Florida Keys.

### Morphometrics

**Figure 2.**
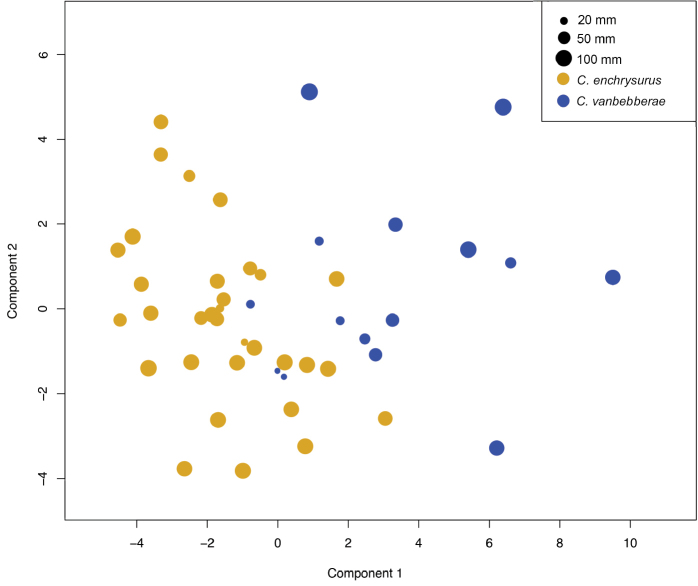
Morphological variation in *Chromisenchrysurus* (yellow) and *Chromisvanbebberae* (blue) specimens, showing PC1 and PC2. Each point represents one individual specimen. Points are scaled according standard length of specimen.

Sixty-eight percent of overall morphometric variation is explained by the first five principal components, of which 29.6% is explained by PC1 (Suppl. material 3: Table S3). Plotting the specimens using scores from PC1 against PC2 separates the two color morphs into well-defined groups (Fig. 2), with areas of overlap consisting primarily of white-tailed individuals smaller than 20 mm SL, suggesting that color morphs may become more distinguished with ontogeny. The strongest loadings in PC1 are, in order of descending absolute value, caudal fin length, longest dorsal soft ray, body depth, and first pelvic soft ray (Suppl. material 4: Table S4). The strongest loadings in PC2 are, in order of descending absolute value, pre-dorsal length, body depth, pre-anal length, and 6^th^ dorsal spine length (Suppl. material 4: Table S4). Yellow-tailed specimens exhibit overall negative scores for component one with a wide range of component two scores, whereas white-tailed specimens exhibit overall positive scores for component one and more positive scores for component two. Many of the individual measurements that contribute substantially to PC1 showed large overlap between the species when looked at individually; however, *C.vanbebberae* sp. nov. does have a significantly longer soft dorsal base (t-test, p = 0.0015), longer last dorsal spines (p = 0.012), longer dorsal rays (p = 2.94e-7), longer anal rays (p = 1.35e-8), a longer caudal-fin (5.597e-8), and longer first pelvic soft rays (p = 0.040).

### Molecular analyses

**Figure 3.**
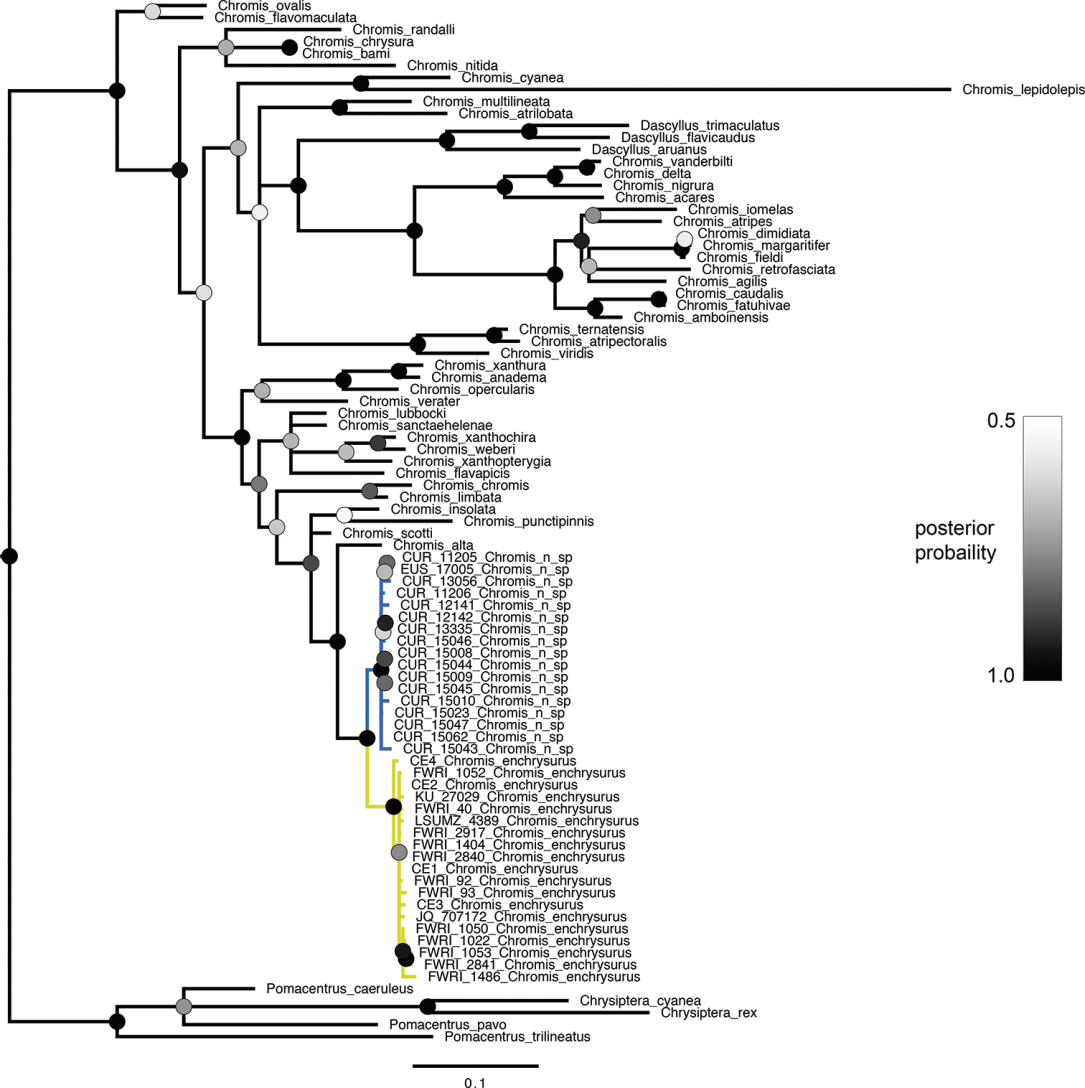
Bayesian phylogenetic analysis of concatenated dataset of pomacentrid species. Circles at nodes indicate posterior probability. Branches with less than 0.50 posterior probability are collapsed. Branch length units are expected number of substitutions per site. Blue and yellow coloring on branches refer to *C.vanbebberae* and *C.enchrysurus*, respectively.

**Figure 4.**
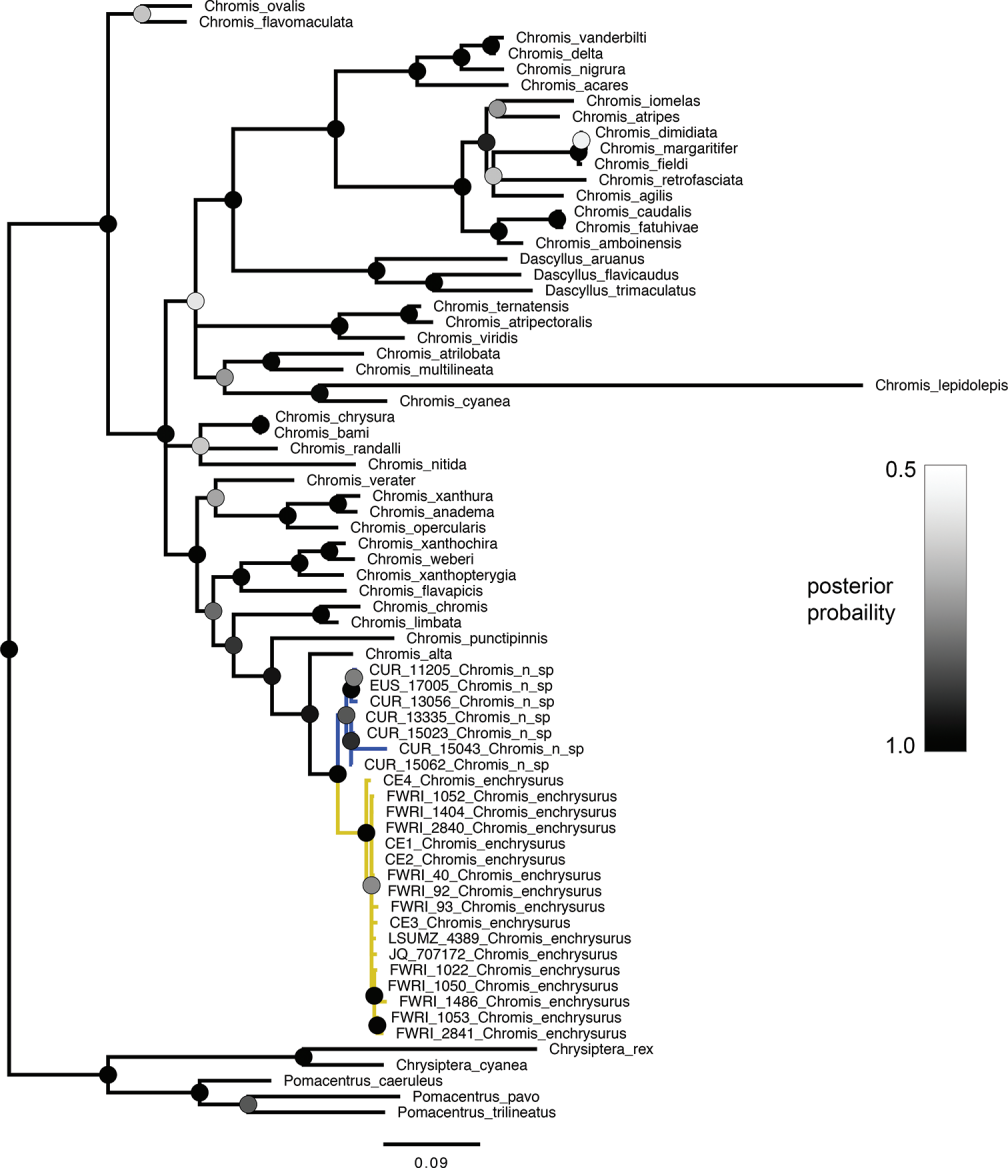
Bayesian phylogenetic analysis of cytb dataset of pomacentrid species. Circles at nodes indicate posterior probability. Branches with less than 0.50 posterior probability are collapsed. Branch length units are expected number of substitutions per site. Blue and yellow coloring on branches refer to *C.vanbebberae* and *C.enchrysurus*, respectively.

**Figure 5.**
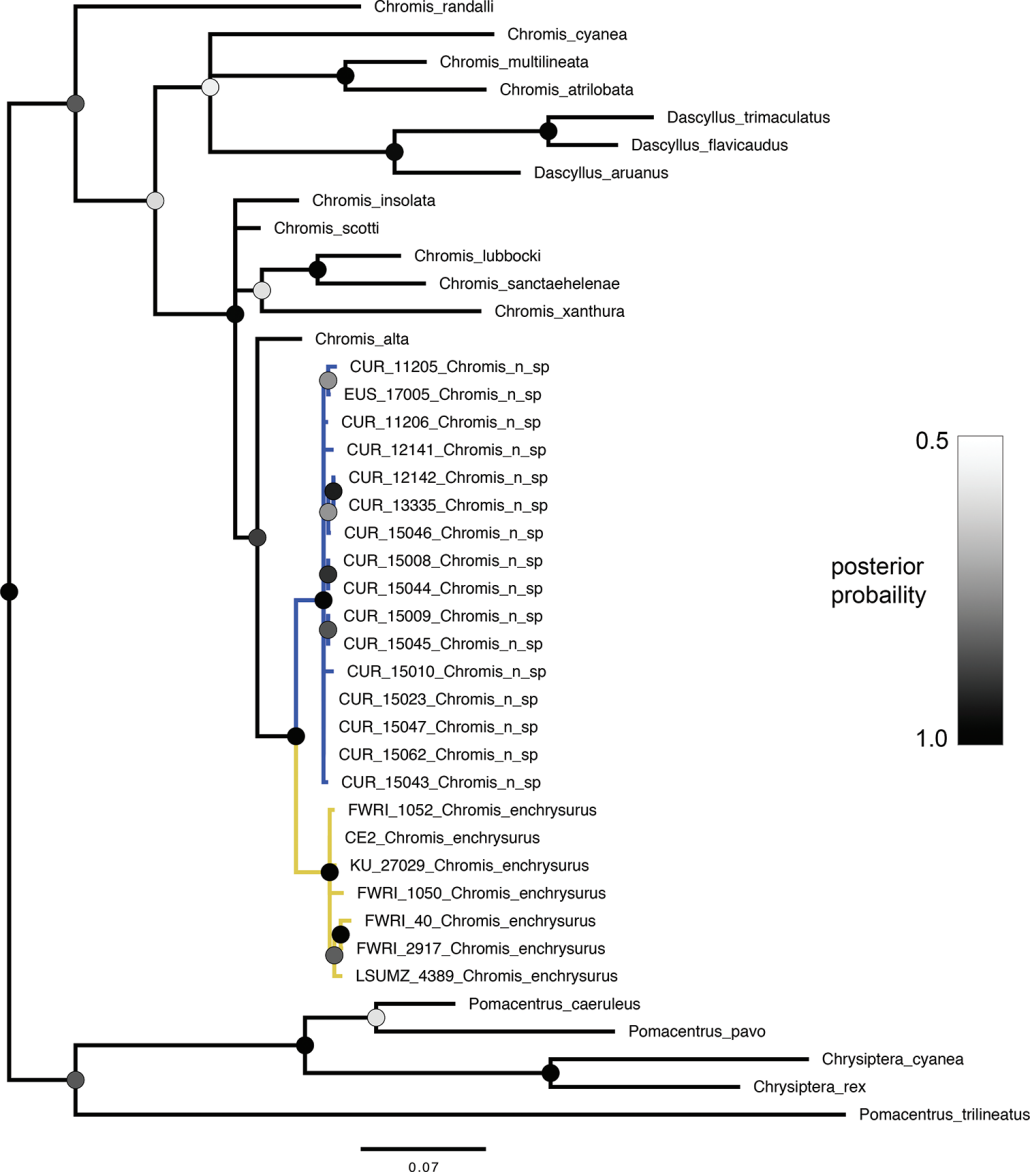
Bayesian phylogenetic analysis of COI dataset of pomacentrid species. Circles at nodes indicate posterior probability. Branches with less than 0.50 posterior probability are collapsed. Branch length units are expected number of substitutions per site. Blue and yellow coloring on branches refer to *C.vanbebberae* and *C.enchrysurus*, respectively.

The individual gene trees and the concatenated tree all recovered the yellow-tailed *Chromis* and white-tailed *Chromis* as reciprocally monophyletic sister taxa. The posterior probability values supporting this relationship are 1.0 in the concatenated tree (Fig. 3) and in both gene trees (Figs 4, 5). Together, the white- and yellow-tailed clade is sister to *C.alta* Greenfield & Woods, 1980, an eastern Pacific species, in all trees (posterior probability = 0.91–0.97).

Analysis of genetic variation between and within groups shows that for both genes assessed, there is substantially more genetic variation between the two color morphs than there is within each. Average pairwise genetic distance in cytb sequences (Table 1) between color morphs was 0.0566, versus 0.0076 within the yellow-tailed group and 0.0218 in white-tailed group. Average genetic distance between the two groups in COI sequences (Table 2) was estimated to be 0.0362, versus 0.0071 within the yellow-tailed group and 0.0042 within the white-tailed group. Taken together, patterns of genetic distance and phylogenetic relationships recovered by the Bayesian phylogenetic analyses support the hypothesis that the two color morphs represent genetically distinct sister species. These genetic differences are corroborated by the morphological differences (Fig. 1) and distinct geographic ranges overlapping in the Florida Keys.

#### Chromis vanbebberae sp. nov.

Taxon classificationAnimaliaTeleosteiPomacentridae

D5F00A3B-5E0D-568C-A5C8-1F311EB280FC

http://zoobank.org/21C7BAA1-2F99-4039-9389-A6069EBC774D

**Figure 6.**
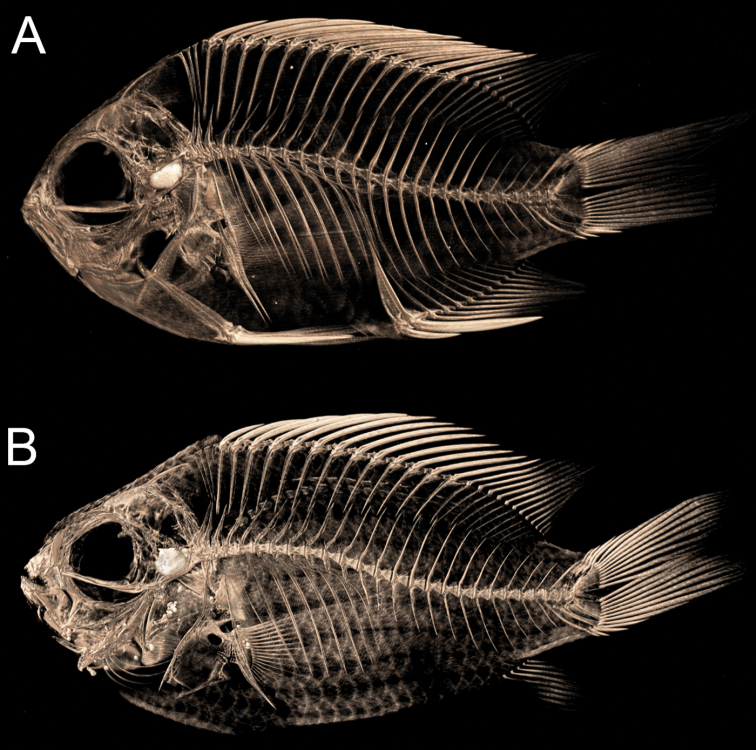
Micro-CT scans **A***Chromisvanbebberae*, Curaçao, paratype, USNM 414901, 33.4 mm SL **B***C.enchrysurus*, South of Marathon, Florida, UW 200011, 41.5 mm SL.

**Figure 7.**
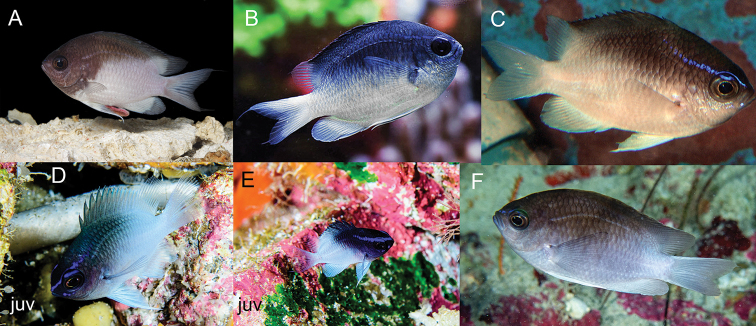
Livecoloration of *Chromisvanbebberae***A** Curaçao, holotype, USNM 446947, 73.9 mm SL **B, C** Curaçao **D, E** St. Paul’s Rocks, Brazil, juveniles **F** St. Paul’s Rocks, Brazil. Photographs by Barry B. Brown (**A**), Yi-Kai Tea (**B**), D. Ross Robertson (**C, D**), Luiz A. Rocha (**E, F**).

**Figure 8.**
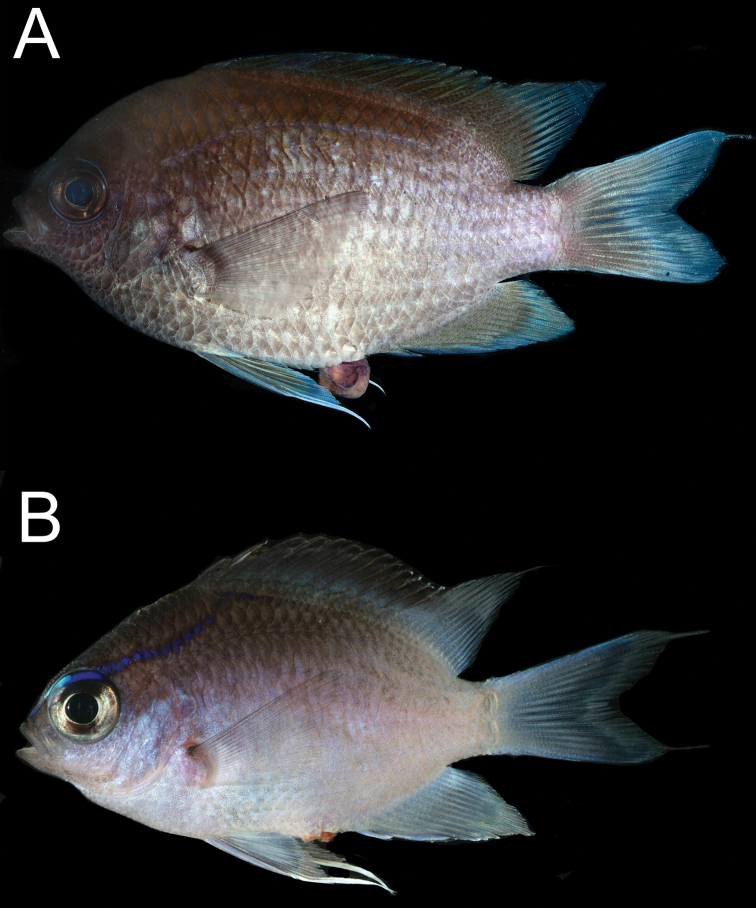
Freshly collected *Chromisvanbebberae***A** paratype, CAS 247234, 90.7 mm SL, Curaçao **B** paratype, USNM 414902, 36.1 mm SL, Curaçao. Photographs by Carole C. Baldwin.

**Figure 9.**
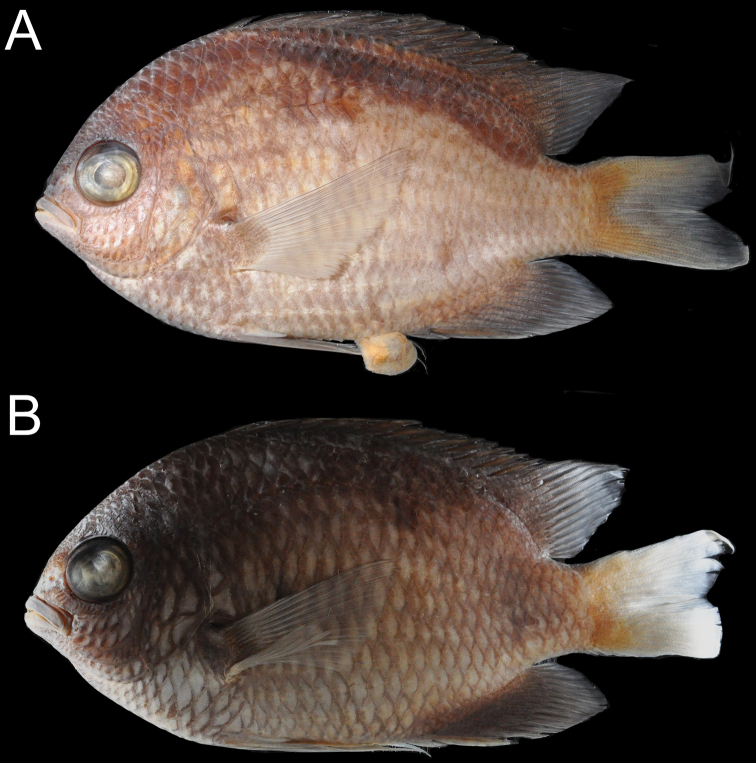
Preserved *Chromisvanbebberae***A** paratype, CAS 247234, 90.7 mm SL, Curaçao **B** paratype, UW 200070, 97.1 mm SL, Curaçao. Photographs by Luke Tornabene.

Whitetail Reeffish Figures 6 , 7 , 8 , 9

##### Type material.

***Holotype.*** USNM 446947, 73.9 mm SL, CURASUB19-01, tissue no. CUR19001, 117 m, Substation Curaçao Downline, Bapor Kibra, Curaçao, 12.0832, -68.8991, C.C. Baldwin, L. Tornabene, B. Van Bebber, W.B. Ludt, 6 May 2019.

***Paratypes*. Curaçao**: All collected at the type locality off Curaçao: USNM 414901, 33.4 mm SL, CURASUB12-15, tissue no. CUR12142, 123–160 m, A. Schrier, B. Brandt, C.C. Baldwin, A. Driskell, P. Mace, 10 Aug 2012; USNM 414902, 36.1 mm SL, CURASUB12-15, tissue no. CUR12141, 123–160 m, A. Schrier, B. Brandt, C.C. Baldwin, A. Driskell, P. Mace, 10 Aug 2012; USNM 413966, 24.7 mm SL, CURASUB13-03, tissue no. CUR13056, 53–189 m, C.C. Baldwin, A. Schrier, D.R. Robertson, C.I. Castilla, B. Brandt, 7 Feb 2013; USNM 413947, 23.4 mm SL, CURASUB13-02, tissue no. CUR13013, C.C. Baldwin, A. Schrier, D.R. Robertson, C.I. Castilla, B. Brandt, 6 Feb 2013; USNM 430030, 14.9 mm SL, tissue no. CUR13335, Substation Curaçao Crew, 9 July 2013; USNM 406206, 24.1 SL, CURASUB11-03, tissue no. CUR11206, 119–161 m, A. Schrier, M. van der Huls, C.C. Baldwin, D.R. Robertson, J. Oliver, 24 May 2011; CAS 247234, 90.7 mm SL, CURASUB19-02, tissue no. CUR19010, C.C. Baldwin, L. Tornabene, T. Christiaan, S. Yerrace, 7 May 2019; UW 200069, 98.4 mm SL, tissue no. CUR19003, 106 m, C.C. Baldwin, L. Tornabene, B. Van Bebber, W.B. Ludt, 6 May 2019; UW 200070, 97.1 mm SL, CURASUB19-02, tissue no. CUR19009, C.C. Baldwin, L. Tornabene, T. Christiaan, S. Yerrace, 7 May 2019; **Sint Eustatius**: USNM 442658, 13.9 mm SL, CURASUB17-17, tissue no. EUS17005, South and southeast of R/V Chapman mooring, SW of island, Kay Bay, St. Eustatius, 17.4599, -62.9817, C.C. Baldwin, L. Tornabene, B. Brandt, J. Casey, 15 April 2017. See Suppl. material 1: Table S1 for non-type material examined.

##### Type locality.

Curaçao, Netherland Antilles.

##### Diagnosis.

Dorsal rays XIII, 12–13; anal rays II, 12–13; pored lateral-line scales 15–18 (usually 17; one paratype with ten and no apparent scale loss or damage); gill rakers 7–8+16–18. Proportional measurements expressed as percent standard length, unless otherwise noted as percent head length (HL): head length 30.2–41.0 (mean 35.4); predorsal length 31.1–42.0 (mean 34.9); orbit diameter 11.5–17.4 (mean 14.6), 39.0 (35.4–48.5) % HL; upper jaw length 9.1 (6.0–14.4), 30.0 (22.3–34.8) % HL; snout length 7.8 (6.9–10.3), 26.0 (17.5–32.2) % HL; interorbital width 10.7 (8.6–12.8), 35.4 (21.1–37.4) % HL; body depth 41.6–57.8 (mean 51.8); caudal length 29.7–44.9 (mean 37.20); last dorsal spine 10.2–16.4 (mean 13); longest dorsal ray 21.1–26.5 (mean 23.3); longest anal ray 18.9–28 (mean 24.3); 1^st^ pelvic soft ray 28.8–43.2 (mean 36.4). See Table 3. Livecoloration with thin iridescent blue oblique stripe extending from snout, through eye, ending below origin of spinous dorsal fin, dorsal half of head dark blue to dusky gray, dark coloration continuing in oblique line across dorsal half of body to end of spinous dorsal fin; ventral half of body, soft dorsal fin, paired fins, and caudal fin white; no yellow pigmentation on body or fins.

##### Description.

***Body*** deep, 55.2 (41.6–57.8), laterally compressed, width 19.4 (16.6–21.6), oval in shape; eyes large, 11.8 (11.5–17.4), interorbital width 10.7 (8.6–12.1). Mouth small, upper jaw length 9.1 (6.0–14.4), terminal, and oblique. Head large, 30.2 (30.2–41.0) and rounded with a convex forehead and short snout 7.8 (5.2–10.3), snout length shorter than orbit diameter (snout ~ 1.8 times in orbit). Preopercle mostly smooth with slight serration at ventral angle; opercle possesses one large spine on dorsal posterior side. Suborbital bones mostly joined to cheek, save for second and third, which flex away from cheek with preorbital. Vertebrae 25 plus urostyle (Fig. 6). Gill rakers very long and slender, closely spaced, equal to or greater than the length of gill filaments, with very fine serrations, 7+17 (7–8+16–18). Teeth in both jaws short and conical, arranged in three rows anteriorly, outer row very slightly enlarged, becoming two rows posteriorly.

***Dorsal fin*** XIII, 12 (12–13); longest dorsal ray 23.8 (21.1–28.5); last (13^th^) dorsal spine 16.4 (10.3–16.4); spinous dorsal base 48.6 (35.5–50.2); soft dorsal base 18.9 (13.4–18.9); pre-dorsal length 33.2 (31.1–42.0). Anal fin II, 12 (12–13); longest anal-fin ray 23.4 (18.9–28.0); pre-anal length 64.1 (63.2–69.0). Pectoral fin 18 (17–20) and lacking free rays; longest pectoral ray 34.2 (31.1–38.1). Pelvic fin I, 5; with a very long first pelvic ray 40.9 (28.8–43.2); pre-pelvic length 35.2 (35.2–43.6). Caudal fin forked with length 41.0 (29.7–44.9).

***Scales*** large, coarsely ctenoid, covering body and most of head, often densely clustered at base of dorsal and anal fins. Pored lateral-line scales 17 (15–18), total scales in lateral series 28 (26–28); one paratype (USNM 430030, 14.9 mm SL) with only 10 pored lateral-line scales, lateral line terminating below the 10^th^ dorsal spine in all individuals, without apparent damage or scale loss. Scales above lateral line 4 (3–4). Scales below lateral line 10 (10–11). Circumpeduncular scales 14 (13–4). No obvious pored or pitted scales on caudal peduncle.

***Livecoloration*** (Fig. 7): Adults (Fig. 7A–C, F) charcoal gray, sometimes tinged with iridescent blue from head to end of spinous dorsal base, with an abrupt, oblique division between dark dorsal portion and light lower body starting at pectoral-fin base and extending to end of spinous dorsal fin; ventral portion of body, soft dorsal fin, paired fins, and caudal fin bright white with no yellow pigmentation. Head with short, oblique iridescent blue stripe originating on upper lip extending through upper edge of eye extending onto side of nape above pectoral fin. In larger individuals, blue stripe reduced, present only on snout. Juvenile (Fig. 7D, E) pigmentation same as adult except dark area distinctly tinged with more blue iridescence and terminating halfway along spinous dorsal fin (versus at end of spinous dorsal fin in adults), blue stripe on head much more prominent, and a second shorter blue stripe often present ventral to eye.

***Coloration in freshly dead specimens*** (Fig. 8): Coloration similar to that of live specimens with little or no blue iridescence except in juveniles, where blue stripe through eye is visible. Paired fins, anal fin, and caudal fin pale to dusky, not vibrant white.

***Coloration in preservation*** (Fig. 9): Base coloration of body pale yellow to golden brown, areas blue or grey in life dark brown; spinous dorsal fin uniformly dark brown, soft dorsal fin, anal fin, and pelvic fin dusky light grey, pectoral fin pale, caudal fin light brown at base becoming pale posteriorly.

##### Distribution

**(Fig. 1).***Chromisvanbebberae* occurs off Bermuda, the Florida Keys, the Bahamas, scattered sites in the northwest, central, eastern and southern Caribbean, and south to at least São Paulo, Brazil, including the offshore islands of Rocas Atoll, St. Paul Rocks, Trindade, and Fernando de Noronha.

##### Habitat.

*Chromisvanbebberae* occurs on a variety of deep-reef habitats at depths between 49 and at least 178 m, including on rocky reef slopes, coral outcroppings, around sponges, boulders, and caves. In areas of colder water in southeastern Brazil (Espírito Santo, Rio de Janeiro and Sao Paulo states) they are seen in depths as shallow as 10 m. In Curaçao, individuals are often found near sporadic patches of rocks located on otherwise open sandy bottoms devoid of other structure, which they frequently co-occupy with the seabasses *Serranusphoebe* or *S.notospilus*. They are also frequently found around artificial substrates such as shipwrecks (e.g., the wreck Queen of Nassau in southeast Florida), tires, and derelict ropes and fishing gear. This species and *C.insolata* Cuvier & Valenciennes, 1830, are the two most common pomacentrids on lower-mesophotic and rariphotic reefs in the Caribbean. In Brazil, *C.insolata* is replaced by its southern mesophotic counterpart, *C.jubauna* Moura, 1995, and the latter often schools with *C.vanbebberae* on coastal reefs; however, *C.vanbebberae* is the only mesophotic *Chromis* recorded in Brazilian oceanic islands.

Where *C.vanbebberae* and *C.enchrysurus* overlap in southeastern Florida, the two species segregate by depth, with *C.enchrysurus* occurring from (~ 25–40 m), and *C.vanbebberae* occurring in deeper water (~ 60–90 m). Emery and Smith-Vaniz (1982) reported a depth range of 5–146 m for *C.enchrysurus*, noting that most observations were from 40–70 m. The 146 m record was from Puerto Rico, and thus represents *C.vanbebberae*, not *C.enchrysurus*. Based on the confirmed records of *C.enchrysurus* from this study, the known depth range of that species is 5–97 m.

##### Etymology.

The species epithet *vanbebberae*, Latinized from Van Bebber, honors Barbara Van Bebber, one of the most accomplished submersible pilots in the Caribbean. Van Bebber was one of several skilled pilots of the ‘Curasub’ that assisted DROP with observations and collections of many new species, including this species. The common name “Whitetail Reeffish” (castañeta coliblanca in Spanish) refers to the caudal-fin coloration that distinguishes the species from *Chromisenchrysurus*, the Yellowtail Reeffish.

##### Remarks.

**Figure 10.**
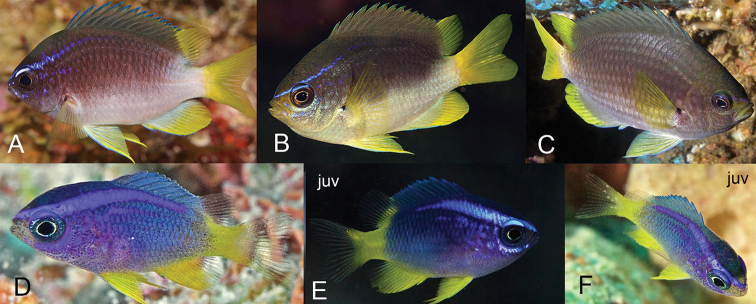
Livecoloration of *Chromisenchrysurus***A** dry Tortugas, Florida **B** off North Carolina **C** gulf of Mexico, Florida **D–F** Florida Keys, juveniles. Photographs by Alison and Carlos Estape (**A, D–F**), Frank Krasovec (**B**), and Bob and Carol Cox (**C**). No photographed fish were preserved.

*Chromisvanbebberae* is easily distinguished from *C.enchrysurus* (Fig. 10) in having white versus yellow on the caudal fin, pelvic fins, anal fin, and posterior rays of the dorsal fin; however, this rapidly fades in death and preservation, making the two nearly indistinguishable. The two species are otherwise morphologically very similar, and species identity of preserved fishes can be most reliably determined based on locality of collection and genetics.

**Figure 11.**
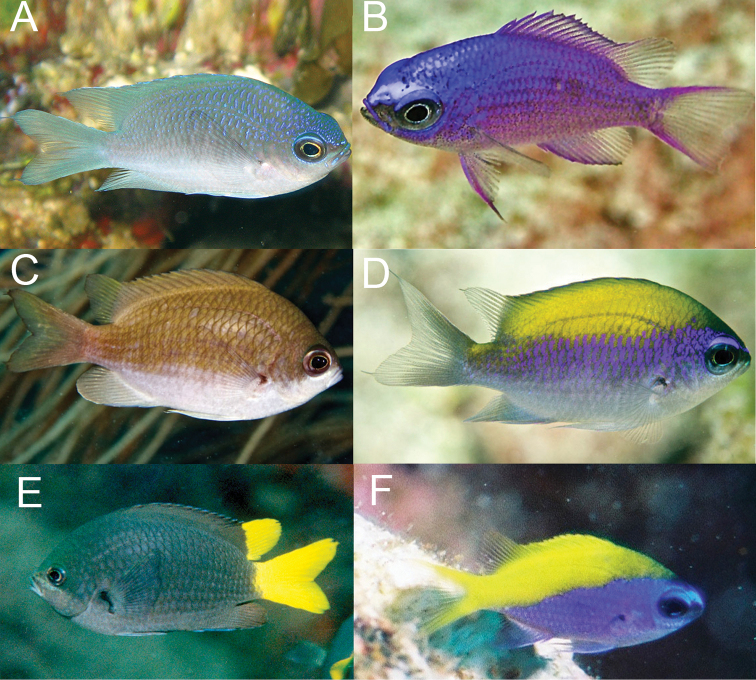
Livecoloration of *Chromisscotti and C.insolata***A***C.scotti*, adult, Roatan, Honduras **B***C.scotti*, juvenile, Tobago **C***C.insolata*, adult, Florida Keys **D***C.insolata*, juvenile, Florida Keys **E***C.jubauna*, adult, Laje de Santos Island, Brazil **F***C.jubauna*, juvenile, Laje de Santos Island, Brazil. Photographs by Mickey Charteris (**A**), Alison and Carlos Estape (**B–D**), and Osmar Luiz Jr (**E, F**).

*Chromisvanbebberae* frequently co-occurs with *C.insolata* and *C.scotti* Emery, 1968, in the Caribbean, and with *C.jubauna* in Brazil. It can be distinguished from *C.scotti* in having an abrupt, diagonal dividing line between the dark dorsal portion of body and white ventral portion of the body (a diffuse horizontal dividing line in *C.scotti*), and in lacking the prominent iridescent light blue coloration that is present on most of the dorsal portion of the body of *C.scotti* (Fig. 11). In addition, the tail is dusky in *C.scotti* versus bright white in *C.vanbebberae*. The diagonal light/dark divide on the body of *C.vanbebberae* also distinguishes it from adult *C.insolata*, which has a horizontal division similar to *C.scotti* (Fig. 11). *Chromisinsolata* and *C.jubauna* both differ from *C.vanbebberae* in number of anal rays: *C.insolata* typically possesses eleven anal rays and *C.jubauna* 9–11, in comparison to the typical 12 (rarely 11 or 13) of *C.vanbebberae*. In addition, *C.insolata* typically possesses 18–19 pored lateral line scales, whereas no *C.vanbebberae* specimens examined exceed 17. Adult *C.jubauna* have uniformly grey to black bodies with bright yellow caudal and soft dorsal fins, versus the dark/light bodies and white fins of *C.vanbebberae*. The juveniles of *C.vanbebberae*, *C.insolata*, *C.scotti*, *C.enchrysurus*, and *C.jubauna* also have dramatically different live coloration (Figs 7, 11). The juveniles of *C.vanbebberae* are similar to adults in coloration, except with slightly more blue iridescence, whereas juvenile *C.scotti* are almost entirely blue, juvenile *C.insolata* have prominent, wide yellow, purple, and white horizontal stripes, and juvenile *C.jubauna* are yellow dorsally and bright purplish-blue ventrally.

## Discussion

Genetic analyses support the hypothesis that yellow-tailed and white-tailed specimens represent distinct species. Bayesian phylogenetic analysis of both genes and of the concatenated sequences returned topologies splitting the two species into reciprocally monophyletic clades with high posterior probability values. Additionally, genetic distance analyses demonstrate that for both genes, sequence variation between species is greater than that within species. While the within-group genetic distance of *C.vanbebberae*cytb sequences is higher than the within-group distance of *C.enchrysurus*, both values are distinctly lower than the between-group variation for the vast majority of species in our analysis (Table 1). In the COI analysis, within-group distance of *C.vanbebberae* is similar to that of *C.enchrysurus*. Both within-group distances of *C.enchrysurus* and *C.vanbebberae* were at least one order of magnitude lower than any between-group value in the analysis (Table 2).

**Table 1.** Average genetic distance in mitochondrial gene cytb between species of Chromis. The number of base differences per site from averaging over alls equence pairs between groups are shown. Average within-species p-distance are shown on the diagonal.

		1	2	3	4	5	6	7	8	9	10	11	12	13	14	15	16	17	18	19	20	21	22	23	24	25	26	27	28	29	30	31	32	33	34	35	36	37	38	39	40	41
1	*C.enchrysurus*	0.008	**0.057**	0.122	0.136	0.119	0.124	0.140	0.146	0.121	0.143	0.169	0.161	0.128	0.158	0.129	0.132	0.141	0.129	0.117	0.177	0.149	0.114	0.142	0.116	0.131	0.178	0.161	0.195	0.140	0.141	0.157	0.132	0.157	0.156	0.139	0.141	0.159	0.151	0.163	0.146	0.079
2	*C.vanbebberae*	**0.057**	0.022	0.128	0.143	0.124	0.136	0.156	0.144	0.135	0.154	0.172	0.182	0.147	0.171	0.157	0.141	0.146	0.138	0.125	0.166	0.154	0.124	0.141	0.138	0.150	0.167	0.182	0.225	0.156	0.134	0.160	0.145	0.160	0.175	0.149	0.161	0.167	0.159	0.147	0.150	0.074
3	*C.verater*	0.122	0.128		0.099	0.074	0.086	0.136	0.142	0.111	0.145	0.139	0.136	0.086	0.154	0.120	0.114	0.139	0.099	0.086	0.146	0.120	0.111	0.146	0.090	0.083	0.145	0.136	0.167	0.136	0.108	0.136	0.114	0.108	0.142	0.105	0.111	0.136	0.136	0.136	0.136	0.117
4	*C.xanthura*	0.136	0.143	0.099		0.114	0.127	0.139	0.176	0.086	0.157	0.164	0.145	0.117	0.167	0.157	0.151	0.157	0.123	0.037	0.152	0.151	0.139	0.178	0.111	0.114	0.154	0.145	0.182	0.139	0.136	0.154	0.123	0.127	0.145	0.139	0.123	0.148	0.154	0.176	0.151	0.127
5	*C.xanthochira*	0.119	0.124	0.074	0.114		0.025	0.142	0.130	0.114	0.154	0.157	0.164	0.096	0.164	0.133	0.123	0.145	0.105	0.105	0.168	0.136	0.114	0.127	0.080	0.093	0.170	0.164	0.198	0.142	0.068	0.179	0.093	0.130	0.160	0.108	0.117	0.167	0.136	0.164	0.157	0.120
6	*C.weberi*	0.124	0.136	0.086	0.127	0.025		0.145	0.136	0.123	0.154	0.170	0.167	0.090	0.167	0.139	0.133	0.148	0.123	0.117	0.175	0.139	0.130	0.140	0.093	0.093	0.170	0.167	0.198	0.145	0.071	0.182	0.108	0.139	0.170	0.117	0.130	0.164	0.142	0.176	0.164	0.127
7	*C.vanderbilti*	0.140	0.156	0.136	0.139	0.142	0.145		0.164	0.130	0.056	0.160	0.136	0.127	0.105	0.127	0.123	0.142	0.133	0.127	0.165	0.151	0.145	0.178	0.142	0.123	0.164	0.139	0.173	0.000	0.164	0.139	0.160	0.157	0.130	0.139	0.133	0.148	0.167	0.164	0.136	0.151
8	*C.ternatensis*	0.146	0.144	0.142	0.176	0.130	0.136	0.164		0.154	0.136	0.176	0.157	0.157	0.179	0.157	0.160	0.093	0.145	0.157	0.171	0.160	0.160	0.175	0.133	0.160	0.173	0.160	0.194	0.164	0.136	0.176	0.170	0.167	0.154	0.157	0.157	0.170	0.043	0.173	0.173	0.145
9	*C.opercularis*	0.121	0.135	0.111	0.086	0.114	0.123	0.130	0.154		0.148	0.179	0.173	0.120	0.145	0.127	0.123	0.148	0.108	0.080	0.165	0.154	0.130	0.124	0.123	0.117	0.164	0.170	0.188	0.130	0.139	0.179	0.142	0.145	0.167	0.133	0.139	0.157	0.151	0.160	0.167	0.123
10	*C.nigura*	0.143	0.154	0.145	0.157	0.154	0.154	0.056	0.136	0.148		0.151	0.133	0.127	0.111	0.142	0.145	0.127	0.139	0.145	0.162	0.151	0.151	0.181	0.130	0.130	0.164	0.136	0.182	0.056	0.160	0.148	0.160	0.167	0.127	0.154	0.145	0.148	0.145	0.160	0.151	0.151
11	*C.iomelas*	0.169	0.172	0.139	0.164	0.157	0.170	0.160	0.176	0.179	0.151		0.099	0.167	0.154	0.194	0.188	0.179	0.157	0.160	0.102	0.151	0.170	0.219	0.157	0.164	0.108	0.096	0.182	0.160	0.164	0.093	0.157	0.160	0.105	0.167	0.148	0.096	0.173	0.086	0.090	0.167
12	*C.dimidiata*	0.161	0.182	0.136	0.145	0.164	0.167	0.136	0.157	0.173	0.133	0.099		0.154	0.154	0.173	0.167	0.160	0.160	0.139	0.105	0.148	0.164	0.213	0.139	0.151	0.108	0.003	0.179	0.136	0.173	0.105	0.148	0.160	0.009	0.157	0.136	0.105	0.154	0.114	0.096	0.160
13	*C.chrysura*	0.128	0.147	0.086	0.117	0.096	0.090	0.127	0.157	0.120	0.127	0.167	0.154		0.148	0.133	0.127	0.148	0.083	0.105	0.146	0.117	0.096	0.162	0.096	0.003	0.145	0.157	0.188	0.127	0.114	0.157	0.099	0.139	0.154	0.090	0.130	0.148	0.139	0.154	0.154	0.133
14	*C.acares*	0.158	0.171	0.154	0.167	0.164	0.167	0.105	0.179	0.145	0.111	0.154	0.154	0.148		0.182	0.160	0.170	0.142	0.160	0.162	0.154	0.148	0.184	0.170	0.145	0.160	0.151	0.160	0.105	0.167	0.160	0.179	0.127	0.154	0.139	0.157	0.157	0.167	0.154	0.176	0.139
15	*C.chromis*	0.129	0.157	0.120	0.157	0.133	0.139	0.127	0.157	0.127	0.142	0.194	0.173	0.133	0.182		0.049	0.139	0.145	0.133	0.194	0.170	0.167	0.156	0.130	0.130	0.191	0.176	0.188	0.127	0.154	0.170	0.167	0.170	0.173	0.139	0.136	0.170	0.151	0.176	0.157	0.151
16	*C.limbata*	0.132	0.141	0.114	0.151	0.123	0.133	0.123	0.160	0.123	0.145	0.188	0.167	0.127	0.160	0.049		0.139	0.123	0.133	0.181	0.145	0.154	0.152	0.120	0.123	0.179	0.170	0.204	0.123	0.154	0.167	0.164	0.151	0.160	0.123	0.139	0.154	0.154	0.170	0.154	0.136
17	*C.viridis*	0.141	0.146	0.139	0.157	0.145	0.148	0.142	0.093	0.148	0.127	0.179	0.160	0.148	0.170	0.139	0.139		0.136	0.148	0.165	0.127	0.142	0.175	0.154	0.151	0.170	0.160	0.170	0.142	0.154	0.148	0.160	0.148	0.154	0.145	0.136	0.148	0.111	0.170	0.160	0.136
18	*C.ovalis*	0.129	0.138	0.099	0.123	0.105	0.123	0.133	0.145	0.108	0.139	0.157	0.160	0.083	0.142	0.145	0.123	0.136		0.117	0.156	0.127	0.077	0.140	0.108	0.080	0.154	0.160	0.204	0.133	0.123	0.164	0.108	0.136	0.154	0.062	0.133	0.160	0.136	0.154	0.157	0.127
19	*C.anadema*	0.117	0.125	0.086	0.037	0.105	0.117	0.127	0.157	0.080	0.145	0.160	0.139	0.105	0.160	0.133	0.133	0.148	0.117		0.133	0.130	0.111	0.162	0.099	0.102	0.136	0.139	0.176	0.127	0.130	0.145	0.117	0.117	0.139	0.127	0.114	0.136	0.142	0.151	0.145	0.114
20	*C.caudalis*	0.177	0.166	0.146	0.152	0.168	0.175	0.165	0.171	0.165	0.162	0.102	0.105	0.146	0.162	0.194	0.181	0.165	0.156	0.133		0.140	0.143	0.197	0.156	0.143	0.006	0.102	0.194	0.165	0.184	0.117	0.162	0.156	0.108	0.146	0.140	0.095	0.168	0.073	0.108	0.162
21	*C.atrilobata*	0.149	0.154	0.120	0.151	0.136	0.139	0.151	0.160	0.154	0.151	0.151	0.148	0.117	0.154	0.170	0.145	0.127	0.127	0.130	0.140		0.117	0.184	0.157	0.120	0.142	0.148	0.179	0.151	0.151	0.154	0.154	0.114	0.148	0.130	0.142	0.160	0.154	0.145	0.154	0.160
22	*C.randalli*	0.114	0.124	0.111	0.139	0.114	0.130	0.145	0.160	0.130	0.151	0.170	0.164	0.096	0.148	0.167	0.154	0.142	0.077	0.111	0.143	0.117		0.140	0.117	0.096	0.145	0.164	0.188	0.145	0.145	0.167	0.114	0.130	0.157	0.086	0.127	0.173	0.145	0.145	0.167	0.114
23	*C.punctipinnis*	0.142	0.141	0.146	0.178	0.127	0.140	0.178	0.175	0.124	0.181	0.219	0.213	0.162	0.184	0.156	0.152	0.175	0.140	0.162	0.197	0.184	0.140		0.152	0.159	0.194	0.213	0.229	0.178	0.162	0.219	0.171	0.184	0.206	0.143	0.171	0.203	0.168	0.181	0.216	0.121
24	*C.flavapicis*	0.116	0.138	0.090	0.111	0.080	0.093	0.142	0.133	0.123	0.130	0.157	0.139	0.096	0.170	0.130	0.120	0.154	0.108	0.099	0.156	0.157	0.117	0.152		0.093	0.151	0.142	0.191	0.142	0.117	0.164	0.117	0.142	0.142	0.108	0.117	0.160	0.127	0.160	0.151	0.123
25	*C.bami*	0.131	0.150	0.083	0.114	0.093	0.093	0.123	0.160	0.117	0.130	0.164	0.151	0.003	0.145	0.130	0.123	0.151	0.080	0.102	0.143	0.120	0.096	0.159	0.093		0.142	0.154	0.185	0.123	0.111	0.154	0.099	0.136	0.151	0.086	0.127	0.145	0.136	0.151	0.151	0.130
26	*C.fatuhivae*	0.178	0.167	0.145	0.154	0.170	0.170	0.164	0.173	0.164	0.164	0.108	0.108	0.145	0.160	0.191	0.179	0.170	0.154	0.136	0.006	0.142	0.145	0.194	0.151	0.142		0.111	0.188	0.164	0.185	0.120	0.164	0.154	0.111	0.145	0.142	0.105	0.170	0.080	0.114	0.157
27	*C.fieldi*	0.161	0.182	0.136	0.145	0.164	0.167	0.139	0.160	0.170	0.136	0.096	0.003	0.157	0.151	0.176	0.170	0.160	0.160	0.139	0.102	0.148	0.164	0.213	0.142	0.154	0.111		0.179	0.139	0.173	0.108	0.148	0.160	0.012	0.157	0.136	0.102	0.157	0.111	0.093	0.160
28	*C.lepidolepis*	0.195	0.225	0.167	0.182	0.198	0.198	0.173	0.194	0.188	0.182	0.182	0.179	0.188	0.160	0.188	0.204	0.170	0.204	0.176	0.194	0.179	0.188	0.229	0.191	0.185	0.188	0.179		0.173	0.213	0.179	0.198	0.188	0.185	0.188	0.179	0.182	0.191	0.198	0.201	0.182
29	*C.delta*	0.140	0.156	0.136	0.139	0.142	0.145	0.000	0.164	0.130	0.056	0.160	0.136	0.127	0.105	0.127	0.123	0.142	0.133	0.127	0.165	0.151	0.145	0.178	0.142	0.123	0.164	0.139	0.173		0.164	0.139	0.160	0.157	0.130	0.139	0.133	0.148	0.167	0.164	0.136	0.151
30	*C.xanthopterygia*	0.141	0.134	0.108	0.136	0.068	0.071	0.164	0.136	0.139	0.160	0.164	0.173	0.114	0.167	0.154	0.154	0.154	0.123	0.130	0.184	0.151	0.145	0.162	0.117	0.111	0.185	0.173	0.213	0.164		0.173	0.120	0.151	0.182	0.136	0.139	0.173	0.142	0.173	0.167	0.136
31	*C.retrofasciata*	0.157	0.160	0.136	0.154	0.179	0.182	0.139	0.176	0.179	0.148	0.093	0.105	0.157	0.160	0.170	0.167	0.148	0.164	0.145	0.117	0.154	0.167	0.219	0.164	0.154	0.120	0.108	0.179	0.139	0.173		0.167	0.160	0.111	0.160	0.151	0.093	0.167	0.111	0.102	0.154
32	*C.nitida*	0.132	0.145	0.114	0.123	0.093	0.108	0.160	0.170	0.142	0.160	0.157	0.148	0.099	0.179	0.167	0.164	0.160	0.108	0.117	0.162	0.154	0.114	0.171	0.117	0.099	0.164	0.148	0.198	0.160	0.120	0.167		0.145	0.151	0.120	0.139	0.167	0.160	0.167	0.145	0.139
33	*C.multilineata*	0.157	0.160	0.108	0.127	0.130	0.139	0.157	0.167	0.145	0.167	0.160	0.160	0.139	0.127	0.170	0.151	0.148	0.136	0.117	0.156	0.114	0.130	0.184	0.142	0.136	0.154	0.160	0.188	0.157	0.151	0.160	0.145		0.160	0.133	0.127	0.151	0.157	0.148	0.167	0.145
34	*C.margaritifer*	0.156	0.175	0.142	0.145	0.160	0.170	0.130	0.154	0.167	0.127	0.105	0.009	0.154	0.154	0.173	0.160	0.154	0.154	0.139	0.108	0.148	0.157	0.206	0.142	0.151	0.111	0.012	0.185	0.130	0.182	0.111	0.151	0.160		0.148	0.136	0.105	0.154	0.120	0.096	0.154
35	*C.flavomaculata*	0.139	0.149	0.105	0.139	0.108	0.117	0.139	0.157	0.133	0.154	0.167	0.157	0.090	0.139	0.139	0.123	0.145	0.062	0.127	0.146	0.130	0.086	0.143	0.108	0.086	0.145	0.157	0.188	0.139	0.136	0.160	0.120	0.133	0.148		0.136	0.157	0.142	0.151	0.173	0.136
36	*C.cyanea*	0.141	0.161	0.111	0.123	0.117	0.130	0.133	0.157	0.139	0.145	0.148	0.136	0.130	0.157	0.136	0.139	0.136	0.133	0.114	0.140	0.142	0.127	0.171	0.117	0.127	0.142	0.136	0.179	0.133	0.139	0.151	0.139	0.127	0.136	0.136		0.142	0.151	0.148	0.127	0.145
37	*C.atripes*	0.159	0.167	0.136	0.148	0.167	0.164	0.148	0.170	0.157	0.148	0.096	0.105	0.148	0.157	0.170	0.154	0.148	0.160	0.136	0.095	0.160	0.173	0.203	0.160	0.145	0.105	0.102	0.182	0.148	0.173	0.093	0.167	0.151	0.105	0.157	0.142		0.157	0.105	0.090	0.157
38	*C.atripectoralis*	0.151	0.159	0.136	0.154	0.136	0.142	0.167	0.043	0.151	0.145	0.173	0.154	0.139	0.167	0.151	0.154	0.111	0.136	0.142	0.168	0.154	0.145	0.168	0.127	0.136	0.170	0.157	0.191	0.167	0.142	0.167	0.160	0.157	0.154	0.142	0.151	0.157		0.157	0.176	0.136
39	*C.amboinensis*	0.163	0.147	0.136	0.176	0.164	0.176	0.164	0.173	0.160	0.160	0.086	0.114	0.154	0.154	0.176	0.170	0.170	0.154	0.151	0.073	0.145	0.145	0.181	0.160	0.151	0.080	0.111	0.198	0.164	0.173	0.111	0.167	0.148	0.120	0.151	0.148	0.105	0.157		0.099	0.148
40	*C.agilis*	0.146	0.150	0.136	0.151	0.157	0.164	0.136	0.173	0.167	0.151	0.090	0.096	0.154	0.176	0.157	0.154	0.160	0.157	0.145	0.108	0.154	0.167	0.216	0.151	0.151	0.114	0.093	0.201	0.136	0.167	0.102	0.145	0.167	0.096	0.173	0.127	0.090	0.176	0.099		0.160
41	*C.alta*	0.079	0.074	0.117	0.127	0.120	0.127	0.151	0.145	0.123	0.151	0.167	0.160	0.133	0.139	0.151	0.136	0.136	0.127	0.114	0.162	0.160	0.114	0.121	0.123	0.130	0.157	0.160	0.182	0.151	0.136	0.154	0.139	0.145	0.154	0.136	0.145	0.157	0.136	0.148	0.160	

**Table 2.** Average genetic distance in mitochondrial gene COI between species of *Chromis*. The number of base differences per site from averaging over all sequence pairs between groups are shown. Average within-species divergences are shown on diagonal.

	*C.enchrysurus*	*C.insolata*	*C.vanbebberae*	*C.scotti*	*C.lubbocki*	*C.xanthura*	*C.randalli*	*C.sanctaehelenae*	*C.multilineata*	*C.alta*
*C.enchrysurus*	0.007	0.070	0.036	0.065	0.110	0.118	0.143	0.104	0.145	0.057
*C.insolata*	0.070		0.066	0.048	0.088	0.111	0.134	0.095	0.129	0.065
*C.vanbebberae*	0.036	0.066	0.004	0.058	0.105	0.106	0.138	0.100	0.142	0.051
*C.scotti*	0.065	0.048	0.058		0.080	0.109	0.134	0.091	0.121	0.053
*C.lubbocki*	0.110	0.088	0.105	0.080		0.124	0.136	0.085	0.143	0.090
*C.xanthura*	0.118	0.111	0.106	0.109	0.124		0.156	0.108	0.143	0.109
*C.randalli*	0.143	0.134	0.138	0.134	0.136	0.156		0.138	0.144	0.139
*C.sanctaehelenae*	0.104	0.095	0.100	0.091	0.085	0.108	0.138		0.156	0.093
*C.multilineata*	0.145	0.129	0.142	0.121	0.143	0.143	0.144	0.156		0.131
*C.alta*	0.057	0.065	0.051	0.053	0.090	0.109	0.139	0.093	0.13	

The genes used in this study are commonly used in phylogenetic and species-delimitation studies in fishes. Mitochondrial genes are especially useful in species identification and phylogenetic reconstruction due to their high number of copies compared to nuclear DNA, lack of recombination, and comparatively fast evolution (Teletchea 2009); however, having independent data from nuclear genes would be beneficial. Broader-scale relationships within the genus and family presented in this study align with those identified in previous phylogenetic analyses of Pomacentridae using both nuclear and mitochondrial DNA, including the recovery of a paraphyletic *Chromis*, with the genus *Dascyllus* nested within it (Jang-Liaw et al. 2002; Quenouille et al. 2004; Cooper et al. 2009).

Although the PCA does separate the two species on the basis of PC1, the morphometric differences are subtle and fail to perfectly separate the two species, especially when individuals are small (SL < 25 mm). While some characters have statistically significant differences between the two species (i.e., the length of soft dorsal base, length of last dorsal spine [p = 0.012], caudal fin length, etc.; see Morphometrics results above), these characters are not discrete, overlap substantially between species, and are not prominent when individuals are small. Collectively, this makes them largely impractical for diagnosing the two species. Coloration remains the most useful morphological character for distinguishing the species. The presence of sister species that are nearly morphologically identical and distinguished primarily by live coloration is increasingly observed in coral-reef fishes (Victor 2015). Unfortunately, this makes it challenging or impossible to retroactively assign species identity for preserved specimens when no data exist for live coloration or genetics. Although color is not always indicative of species-level differences between closely related reef-fish taxa (Dibattista et al. 2012; Schultz et al. 2007), live color is often the primary, or in some cases only, external character by which species can be distinguished (Luiz et al. 2009; Randall and Rocha 2009). Such differentiating characters are particularly troublesome for distinguishing species of deep-reef fishes, as for centuries, many were seldom observed live and, until recently, none had been sampled genetically.

Data suggest that *C.vanbebberae* and *C.enchrysurus* occupy distinct geographic ranges with little overlap, which indicates that collection locality can help inform species identity with reasonable certainty when genetic analysis cannot be performed. Species-range estimates of *C.enchrysurus* and *C.vanbebberae* based on collections, visual observations, and genetic data from georeferenced specimens agree well with the findings of Robertson and Cramer (2014) on biogeographic patterns and species distributions in the Greater Caribbean. Robertson and Cramer (2014) divided the region into three provinces, each with its own faunal assemblage: a northern province encompassing the Gulf of Mexico and southeastern United States; a central province encompassing the West Indies, Bermuda, and Central America; and a southern province encompassing northern South America. In the Greater Caribbean, the southernmost locality of specimens examined in this study was Curaçao, which falls into the central province, although many photographic records and specimens identified as *C.enchrysurus* exist from the Venezuelan coast and the east coast of South America as far south as Brazil. Thus, *C.enchrysurus* occupies the northern province and *C.vanbebberae* occupies the central and southern provinces of the Greater Caribbean plus Brazil.

A genetic break between sister species or populations occurring in the northern province of Robertson and Cramer (2014; i.e., Gulf of Mexico, eastern U.S.) and those occurring in the Caribbean or South America is a common phylogeographic pattern (Floeter et al. 2008). For example, sister species of *Liopropoma* basslets demonstrate a similar split: *L.eukrines* inhabits the Gulf of Mexico and the Atlantic coast of the southeastern U.S., and *L.aberrans* inhabits the Caribbean (Baldwin and Robertson 2014). Populations of *Bathygobiussoporator* from the Gulf of Mexico and eastern U.S. have also been shown to be distinct from those in the Caribbean and Brazil (Tornabene et al. 2010; Tornabene and Pezold 2011; Rodríguez-Rey et al. 2017). Other examples of sister lineages occurring in the Caribbean versus the Gulf of Mexico/eastern U.S. can be found in the *Menticirrusamericanus* species complex (Marceniuk et al. 2020), the *Lutjanuscampechanus* and *L.purpureus* species pair (Pedraza-Marrón et al. 2019; da Silva et al. 2020), the *Scartellacristata* species complex (Araujo et al. 2020), the genus *Bagre* (Betancur-R 2009), and in *Epinephelusadscensionis* (Carlin et al. 2003). In many cases these speciation patterns are thought to be a product of environmental variation between provinces as opposed to hard barriers to gene flow between the regions (Rocha et al. 2005; Robertson and Cramer 2014). The northern province is a heterogenous, more temperate environment, whereas the central and southern provinces are both more uniform and stable. The central and southern provinces are also more similar to one another than to the northern province, despite the northern and southern provinces bearing similarities in eutrophication and upwelling. We did not have genetic samples from Brazil, and while photographs of *C.vanbebberae* appear similar to those from the Caribbean, it is possible that additional genetic breaks may occur near the Amazon outflow, or between mainland Brazil and off-shore islands (Joyeux et al. 2001; Floeter et al. 2008).

Many of the recently described species from the Greater Caribbean are cryptobenthic fishes that are often overlooked in biodiversity surveys. However, pomacentrids are some of the most conspicuous fishes on corals reefs. They occur on shallow and deep coral reefs in every geographic region, where they are often the most abundant fishes on a given reef (Quenouille et al. 2004). Thus, it may be surprising that two common species that can easily be distinguished when alive and occupy separate ranges have been thought to be the same species for decades. This almost certainly represents a gap in knowledge attributed to a lack of genetic data, coupled with the challenges of observing live fishes below the depth limit of safe conventional SCUBA diving, and the fact that these species are morphologically conserved. Such gaps can result in an underestimation of the overall biodiversity in reef systems. Although reef-fish assemblages on deep and shallow reefs typically come from the same set of families, deep-reef assemblages are taxonomically distinct from shallow reefs at the species level and contain a wealth of previously unknown biodiversity that is still being uncovered (Baldwin et al. 2018; Rocha et al. 2018). Many undescribed species discovered on deep reefs are immediately recognizable as being new to science; however, there are other instances where a single deep-reef species that was described many years ago is revealed to be a complex of two or more species. For example, two new deep-reef basses previously thought to be *Liopropomaaberrans*, which was described in 1860, have since been described as new, splitting that species into three (Baldwin and Johnson 2014; Baldwin and Robertson 2014). Collection of fresh specimens, tissues, and photographs from deep reefs also led to the discovery that individuals previously thought to be juvenile color morphs of the grammatid basslet *Lipogrammaevides* was in fact a distinct species, *L.levinsoni*, with the two species segregating by depth in areas of geographic overlap (Baldwin et al. 2016a). These examples, including the current study, highlight the importance of initiatives that document the fauna of deep reefs through collection of multiple types of data (i.e., photographs, specimens, tissue samples, habitat and depth data, etc.) to gain a more complete understanding of tropical marine biodiversity.

**Table 3.** Morphometrics and meristics of *Chromisvanbebberae* and *Chromisenchrysurus* specimens examined. Morphometric values are as percentage of SL.

	*Chromisvanbebberae*			*Chromisenchrysura*		
	Holotype USNM 446947	Average	Range	Holotype KU 27029	Average	Range
standard length	73.9	48.2	13.9–98.4	68	60.7	80.8–17.7
body depth	55.2	51.1	41.6–57.7	50.9	50.2	53.9–44
body width	19.4	19.1	16.5–21.6	17.8	17.5	19.2–13.8
head length	30.2	35.4	30.2–41	31.3	31.6	36–29.8
snout length	7.9	8.2	5.2–10.3	8.4	8.2	9.3–5.8
orbit diameter	11.8	14.6	11.5–17.4	11.3	11.7	14.7–10
interorbit width	10.7	10.6	8.6–12.1	10.6	10.6	14–9.2
caudal peduncle depth	16.1	15.1	13.3–16.4	14.7	14	15.6–9.8
upper jaw length	9.1	10	6.0–14.4	9.4	9.7	10.9–8
predorsal length	33.2	34	28.6–42	35.6	33.7	38.3–28.2
spinous dorsal base	48.6	44.1	35.5–50.2	45.9	46.7	50.8–36.6
soft dorsal base	18.9	16.5	13.4–18.9	16.9	14.6	18–10.4
1^st^ dorsal spine	8.7	9.1	7.2–11.9	10.3	8.3	10.3–6.7
2^nd^ dorsal spine	12.9	14.3	11.4–17.5	14.9	12.6	16.2–10.6
3^rd^ dorsal spine	15.7	17.9	15.3–21.6	19.6	15.5	19.6–12.3
4^th^ dorsal spine	19.4	20.2	16.6–24.5	22.4	17.4	22.4–13.5
5^th^ dorsal spine	20.6	20.5	16.2–25.9	22.2	17.4	22.4–13.5
6^th^ dorsal spine	19.8	18.6	15.5–23.7	21.6	17	21.6–13.3
last dorsal spine	16.4	13.8	10.3–17.4	14.1	12.3	16.1–9.3
longest dorsal ray	23.8	23.2	21.1–28.5	21.3	19.1	23–16.1
preanal length	64.1	67	63.2–69.7	64.7	66.5	69.9–63.1
1^st^ anal spine	9.3	8.7	5.8–11.6	9.9	8.1	9.9–5.5
2^nd^ anal spine	19.9	19.2	15.1–22.4	20.9	18.8	21.8–16
longest anal ray	23.4	24.1	18.9–28	22.1	19.9	26.3–16.3
caudal length	41	36.8	29.7–44.9	31.5	31.4	35.8–27.3
longest pectoral ray	34.2	33.8	31.1–38.1	30.2	31.2	33.7–28.6
prepelvic length	35.2	38.4	35.2–43.6	37.4	37.3	41.7–33.8
pelvic spine length	22.2	20.3	18.7–22.4	22.7	20	31.2–17.2
1^st^ pelvic soft ray	40.9	35.4	28.8–43.2	36.3	23.4	36.8–30.8
dorsal rays	12	12.73	12–13	12	12.22	11–15
anal rays	12	12.57	12–13	12	12.06	11–13
pored lateral line scales	17	16.54	15–17	17	17.16	16–18
upper gill rakers	7	7.27	7–8	8	7.47	7–8
lower gill rakers	17	16.93	16–18	16	16.84	16–18

## Supplementary Material

XML Treatment for Chromis vanbebberae

## Supplementary materials

Supplementary material 1 Table S1. Material examined of *Chromisvanbebberae* and *C.enchrysurus* Data type Specimen list https://binary.pensoft.net/file/492907This dataset is made available under the Open Database License (http://opendatacommons.org/licenses/odbl/1.0/). The Open Database License (ODbL) is a license agreement intended to allow users to freely share, modify, and use this Dataset while maintaining this same freedom for others, provided that the original source and author(s) are credited.Emily P. McFarland, Carole C. Baldwin, D. Ross Robertson, Luiz A. Rocha, Luke Tornabene

Supplementary material 2 Table S2. GenBank accession numbers for outgroup taxa Data type Accession numbers https://binary.pensoft.net/file/492908This dataset is made available under the Open Database License (http://opendatacommons.org/licenses/odbl/1.0/). The Open Database License (ODbL) is a license agreement intended to allow users to freely share, modify, and use this Dataset while maintaining this same freedom for others, provided that the original source and author(s) are credited.Emily P. McFarland, Carole C. Baldwin, D. Ross Robertson, Luiz A. Rocha, Luke Tornabene

Supplementary material 3 Table S3. Contribution to overall variance by the first ten principle components Data type Principal component contributions https://binary.pensoft.net/file/492909This dataset is made available under the Open Database License (http://opendatacommons.org/licenses/odbl/1.0/). The Open Database License (ODbL) is a license agreement intended to allow users to freely share, modify, and use this Dataset while maintaining this same freedom for others, provided that the original source and author(s) are credited.Emily P. McFarland, Carole C. Baldwin, D. Ross Robertson, Luiz A. Rocha, Luke Tornabene

Supplementary material 4 Table S4. Loadings for the first five components from the Principal Component Analysis Data type Principal component loadings Explanation note Values in blue contribute positively and those in red contribute negatively to a given component. The greater the absolute value of the contribution, the more intense the color. Only the first five of 28 components have been included. https://binary.pensoft.net/file/492910This dataset is made available under the Open Database License (http://opendatacommons.org/licenses/odbl/1.0/). The Open Database License (ODbL) is a license agreement intended to allow users to freely share, modify, and use this Dataset while maintaining this same freedom for others, provided that the original source and author(s) are credited.Emily P. McFarland, Carole C. Baldwin, D. Ross Robertson, Luiz A. Rocha, Luke Tornabene
