# Corrigenda: A new species of *Chromis* damselfish from the tropical western Atlantic (Teleostei, Pomacentridae). ZooKeys 1008: 107–138. https://zookeys.pensoft.net/article/58805/

**DOI:** 10.3897/zookeys.1036.65739

**Published:** 2021-05-10

**Authors:** Emily P. McFarland, Carole C. Baldwin, David Ross Robertson, Luiz A. Rocha, Luke Tornabene

**Affiliations:** 1 School of Aquatic and Fishery Sciences, University of Washington, Seattle, WA 98195-5020, USA University of Washington Seattle United States of America; 2 Burke Museum of Natural History and Culture, Seattle, WA 98105, USA Burke Museum of Natural History and Culture Seattle United States of America; 3 Department of Vertebrate Zoology, National Museum of Natural History, Smithsonian Institution, Washington, DC, 20560, USA Smithsonian Institution Washington United States of America; 4 Smithsonian Tropical Research Institute, Balboa, Republic of Panama Smithsonian Tropical Research Institute Balboa Panama; 5 Department of Ichthyology, California Academy of Sciences, San Francisco, California 94118, USA California Academy of Sciences San Francisco United States of America

## Abstract

N/A

Following the publication of our paper (McFarland et al. 2020), it was brought to our attention by Andrew Bentley (University of Kansas) that Table 3 listed the holotype of the species *Chromisenchrysurus* Jordan & Gilbert, 1882 as KU 27029, and mislabeled the species as *Chromisenchrysura*. We regret this mistake, as there are three syntypes of *C.enchryurus*, none of which is the specimen listed. The corrected version of the table is provided here.

**Table 3.** Morphometrics and meristics of *Chromisvanbebberae* and *Chromisenchrysurus* specimens examined. Morphometric values are as percentage of SL.

	*Chromisvanbebberae*			*Chromisenchrysurus*	
	Holotype of *Chromisvanbebberae* USNM 446947	Average	Range	Average	Range
standard length	73.9	48.2	13.9–98.4	60.7	80.8–17.7
body depth	55.2	51.1	41.6–57.7	50.2	53.9–44.0
body width	19.4	19.1	16.5–21.6	17.5	19.2–13.8
head length	30.2	35.4	30.2–41.0	31.6	36.0–29.8
snout length	7.9	8.2	5.2–10.3	8.2	9.3–5.8
orbit diameter	11.8	14.6	11.5–17.4	11.7	14.7–10.0
interorbit width	10.7	10.6	8.6–12.1	10.6	14.0–9.2
caudal peduncle depth	16.1	15.1	13.3–16.4	14.0	15.6–9.8
upper jaw length	9.1	10.0	6.0–14.4	9.7	10.9–8.0
predorsal length	33.2	34.0	28.6–42.0	33.7	38.3–28.2
spinous dorsal base	48.6	44.1	35.5–50.2	46.7	50.8–36.6
soft dorsal base	18.9	16.5	13.4–18.9	14.6	18.0–10.4
1st dorsal spine	8.7	9.1	7.2–11.9	8.3	10.3–6.7
2nd dorsal spine	12.9	14.3	11.4–17.5	12.6	16.2–10.6
3rd dorsal spine	15.7	17.9	15.3–21.6	15.5	19.6–12.3
4th dorsal spine	19.4	20.2	16.6–24.5	17.4	22.4–13.5
5th dorsal spine	20.6	20.5	16.2–25.9	17.4	22.4–13.5
6th dorsal spine	19.8	18.6	15.5–23.7	17.0	21.6–13.3
last dorsal spine	16.4	13.8	10.3–17.4	12.3	16.1–9.3
longest dorsal ray	23.8	23.2	21.1–28.5	19.1	23.0–16.1
preanal length	64.1	67.0	63.2–69.7	66.5	69.9–63.1
1st anal spine	9.3	8.7	5.8–11.6	8.1	9.9–5.5
2nd anal spine	19.9	19.2	15.1–22.4	18.8	21.8–16.0
longest anal ray	23.4	24.1	18.9–28.0	19.9	26.3–16.3
caudal length	41.0	36.8	29.7–44.9	31.4	35.8–27.3
longest pectoral ray	34.2	33.8	31.1–38.1	31.2	33.7–28.6
prepelvic length	35.2	38.4	35.2–43.6	37.3	41.7–33.8
pelvic spine length	22.2	20.3	18.7–22.4	20.0	31.2–17.2
1st pelvic soft ray	40.9	35.4	28.8–43.2	23.4	36.8–30.8
dorsal rays	12	12.7	12–13	12.2	11–15
anal rays	12	12.6	12–13	12.1	11–13
pored lateral line scales	17	16.5	15–17	17.2	16–18
upper gill rakers	7	7.3	7–8	7.5	7–8
lower gill rakers	17	16.9	16–18	16.8	16–18
