# Supplementary material for: A new species of Chromis damselfish from the tropical western Atlantic (Teleostei, Pomacentridae)
Source: Zookeys. 2020 Dec 31;1008:107–38. doi: 10.3897/zookeys.1008.58805 (PMC7790814; doi:10.3897/zookeys.1008.58805)
Supplement: Supplementary material 3 — Table S3. Contribution to overall variance by the first ten principle components [file zookeys-1008-107-s003.doc]

**Supplementary Table S3. Contribution to overall variance by the first 10 principle components.**

| **Component** | **Proportion** |
| --- | --- |
| 1 | 0.296 |
| 2 | 0.129 |
| 3 | 0.106 |
| 4 | 0.083 |
| 5 | 0.066 |
| 6 | 0.057 |
| 7 | 0.044 |
| 8 | 0.035 |
| 9 | 0.038 |
| 10 | 0.024 |
